# Thyrolipoma presentation as a huge multinodular goiter; A case report of an extremely rare entity

**DOI:** 10.1016/j.ijscr.2023.108936

**Published:** 2023-10-10

**Authors:** Saqer Alenezi, Athary Saleem, Omar Alhajri, Ous Alozairi

**Affiliations:** Department of General Surgery, Al-Adan Hospital, Kuwait

**Keywords:** Lipomatosis, Thyroid adipose metaplasia, Multinodular goiter, Adeolipoma, Total thyroidectomy, Case report

## Abstract

**Importance and importance:**

Thyroid lipomatosis is a rare entity of thyroid gland lesions. The exact etiopathogenesis of this condition is unknown. Most patients presented with compression symptoms. Radiological investigations such as neck ultrasonography (U/S) and computed tomography (CT) are crucial to evaluate and diagnose fat-containing thyroid tumors, while the definitive diagnosis is achieved by the histopathological study.

**Case presentation:**

A 78-year-old female patient, with a background medical history of diabetes mellitus type II and chronic kidney disease, presented to our hospital with a seven-month history of large-sized neck swelling. On physical examination, the neck mass was firm, nodular, hard in consistency, and asymmetrical. The neck swelling was associated with swallowing difficulties and minimal voice changes. Laboratory investigations were unremarkable. Neck U/S showed thyroid goiter. FNA and FNAC were also done. Then, neck CT was performed, and bilateral lobulated fat density was detected. So, a total thyroidectomy was performed, and the resected specimen was sent for histopathology studies. The postoperative period was uneventful.

**Clinical discussion:**

Diffuse thyroid lipomatosis is an unusual non-neoplastic lesion. The clinical features of thyro-lipomatosis include compression symptoms. Radiological tools and cytology aid in diagnosis demonstration but the specific diagnosis is achieved by histopathology.

**Conclusion:**

Due to the rare etiologic origin and unknown pathogenesis of thyrolipoma, we report the case of a 78-year-old female patient with enlarged neck swelling, found to be thyroid lipomatosis.

## Highlights

• Among thyroid gland lesions, thyroid lipomatosis is a rare occurrence. This condition is unknown to have a specific etiopathogenesis.
• There are two types of thyroid lesions containing fat. One of them is fat-containing thyroid neoplasms and the other is non-neoplastic conditions with adipose tissue.
• The clinical features of thyrolipoma include compression symptoms.
• Radiological studies with cytology support the diagnosis but the goal standard in diagnosis thyroid lipomatosis by histopathology.

## 1 Introduction

Thyroid gland tumors that incorporate fat are highly uncommon [[1], [2], [3]]. These thyroid lesions can be classified into two groups: thyroid neoplastic and non-neoplastic lesions. The neoplastic fat-containing lesions involve follicular adenoma, follicular carcinoma, and papillary carcinoma while the non-neoplastic lesions include many other conditions [3]. These include diffuse lipomatosis, amyloid goiter, Graves' disease, and parathyroid lipoma for instance [3,4].

In 1942, Dhayagude described diffuse lipomatosis, a type of fat deposition, for the first time [1]. Diffuse lipomatosis of the thyroid is an extremely rare condition characterized by significant mature adipose tissue infiltration of thyroid parenchyma with the absence of accumulated fibrils of amyloid [5].

Here we report a 78-year-old female patient who presented with a large-sided neck mass that was found to be diffuse thyroid lipomatosis. Our work has been reported in line with the SCARE Guidelines 2020 criteria [6].

## 2 Case presentation

A 78-year-old female patient, with a background medical history of diabetes mellitus type II and chronic kidney disease, presented to our hospital with a seven-month history of large-sized neck swelling. The patient was conscious, alert, oriented, and vitally stable on physical examination. Upon palpation, the neck mass was firm, nodular, hard in consistency, and asymmetrical. The neck swelling was associated with swallowing difficulties and minimal voice changes. There was no lymphadenopathy or other systemic manifestations.

The laboratory investigations on admission are shown in Table 1.

**Table 1:** Patient's laboratory investigations on admission.

Patient's vital signs	Hematology	Biochemistry	Coagulation	Thyroid function tests
GCS 15	WBC 7.9	ALT 12	PT 17.5	TSH 0.261
BP 132/75 mmHg	Hb 92	AST 14	PT-INR 1.24	Free T4 13.9
HR 89 bpm	Plt 321	ALP 118	APTT 32.60	
RR 20 bpm	Neutrophils 4.32	CL 111	APTT ratio 1.19	
SPO2 98 % in room air	Lymphocytes 3.09	Albumin 39.8	–	
Body temperature 37 °C	Monocytes 0.32	Corrected Ca 2.39	–	–
Glucose 8.8	Eosinophils 0.14	Na 143	–	–
Calc osmolarity 306	Basophils 0.03	K 4.3	–	–

**Fig. 1:**
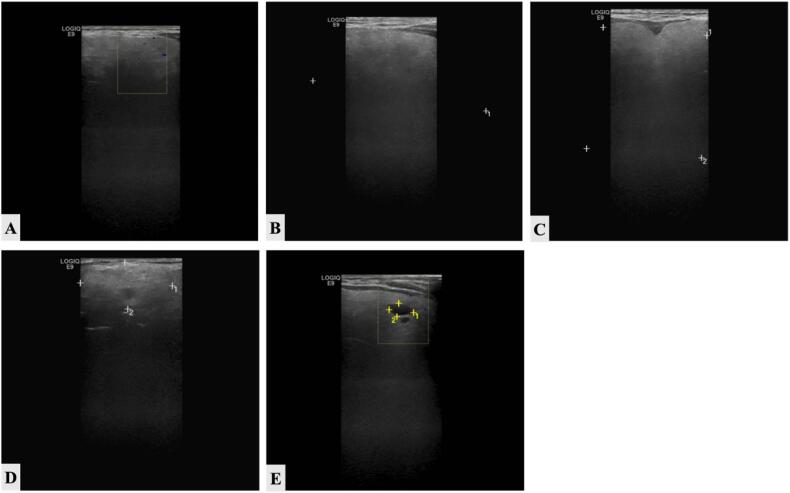
The ultrasound images of the thyroid gland. A, B, C, D, and E showed the ultrasound findings.

The ultrasonography of the neck was performed, showing thyroid goiter with the following findings (Fig. 1):

• Both thyroid gland lobes are diffusely enlarged in size with uniform echotexture (Fig. 1A).
• The right lobe dimension was 8.5 × 6.2 × 5 cm (Fig. 1B).
• The left lobe dimension was 8 × 7.5 × 7.4 cm (Fig. 1C).
• The isthmus size was 1.2 cm.
• The upper aspect of the left thyroid lobe was well well-defined smooth isoechoic solid nodule with no calcification, measuring about 4 × 2 cm (Fig. 1D).
• The right thyroid lobe showed two small cysts measuring 1 and 6 mm and another cyst on the left side measuring 6 mm (Fig. 1E).
• No abnormalities or focal lesions were detected on carotid vessels, jugular veins, trachea, parotid, and submandibular glands.
• The left thyroid lobe solid nodule following TIRAD 3.

Furthermore, the fine needle aspiration (FNA) revealed a left-sided thyroid nodule. As a result, fine needle aspiration cytology (FNAC) was decided but the received aspirate was hemorrhagic and suboptimal for cytological diagnosis.

**Fig. 2:**
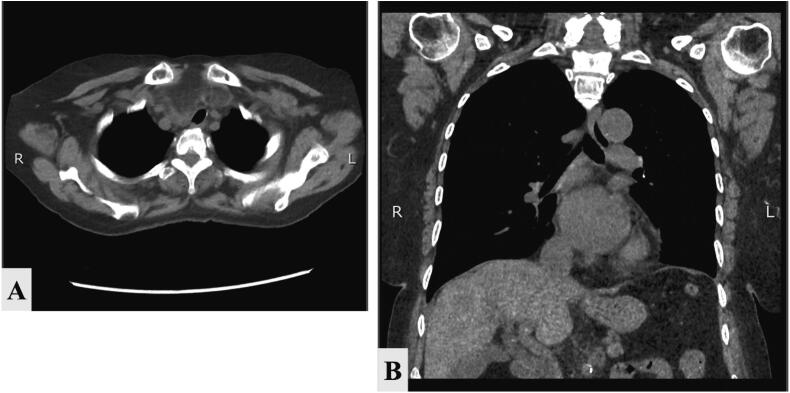
A: Preoperative CT scan showing minimal retrosternal extension of the multinodular goiter. **A:** Axial view. **B:** coronal view.

On the CT scan of the chest, a bulky thyroid gland was noted with bilateral lobulated hypodense lesion and fat density, replacing most of both thyroid lobes with internal septation and minor elements of soft tissue density with evidence of retrosternal extension notably from the right lobe with mild narrowing of the trachea with left shift displacement (Fig. 2).

At this point, a total thyroidectomy was performed. During this, multinodular goiter was detected with minimal retrosternal extension besides the friable fatty tissue that was mainly localized in the right thyroid lobe.

**Fig. 3:**
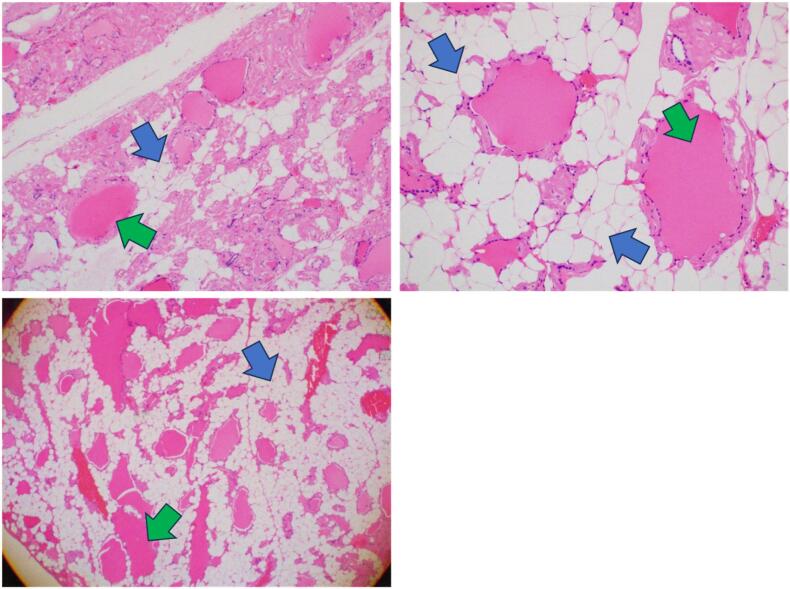
The miscroscopic examination of the resected thyroid tissue showing mature adipocytes (Blue arrow) and distended thyroid follicles (Green arrow). (For interpretation of the references to colour in this figure legend, the reader is referred to the web version of this article.)

Grossly, the resected total thyroid tissue consisted of grey-brownish nodular tissue weighing 300 mg, measuring 22.4 × 8.5 × 5 cm, associated with a grey-whitish cut surface and small cystic spaces. The histopathology study of the resected thyroid tissue revealed mature adipocytes, accounting for 70 % of the volume of the tissue, with distended thyroid follicles that were filled with abundant colloid material and lined with cuboidal epithelial cells. Also, the stroma showed abundant mature adipocytes and fibro-collagenous tissue with no evidence of atypia (Fig. 3).

The postoperative period was uneventful, and the patient was discharged on the fifth postoperative day.

## 3 Discussion

Thyroid lipomatosis is a rare condition that mainly affects the middle-aged population with no gender preference [7]. In thyroid lipomatosis, the thyroid gland is enlarged due to the growth of mature fat cells that are intermixed with thyroid follicles and surrounded by a thin layer of tissue, due to the absence of encapsulation [8].

In the thyroid gland, mature adipose tissue containing lesions include liposarcomas, lipid-rich cell adenomas, and parathyroid or thymic lipomas [9]. Due to the progressive growth rate, malignant lesions should be considered anaplastic carcinoma and thyroid lymphoma [10] The microscopic features of adenolipomas and diffuse thyroid lipomatosis, two infrequent malignancies, include the simultaneous existence of adipocytes and thyroid follicles [3].

Pathophysiologically, the exact mechanism is unknown [10,11]. Several authors suggested multiple theories in order to explain the mechanism of diffuse adipose tissue proliferation. According to a number of authors, the thyroid gland encases clusters of adipocytes during embryogenesis. On the other hand, other researchers suggested an underlying hypoxic pathology [10]. Lau *et al* highlighted a potential link between thyroid lipomatosis and the aberrant differentiation of adipose tissue caused by a mutation in the mitochondrial protein succinate dehydrogenase-subunit B [10].

It is essential to emphasize that these lesions' rapid development and dimensions demand a thorough evaluation right away in order to rule out cancerous conditions such as thyroid lymphoma and anaplastic carcinoma [10].

The clinical manifestations of patients with thyroid lipomatosis are mainly compressive symptoms that result from enlarged thyroid tissue. These include dysphagia, dyspnea, voice alterations, and respiratory difficulties [10]. Our patient presented with compression symptoms, including swallowing difficulties and voice changes.

Initially, the clinical evaluation of thyroid lipomatosis relies on physical examination that usually shows a soft, non-tender goiter that is nodular or diffuse. In most cases, tests show normal thyroid function, but both hyperthyroidism and hypothyroidism have been described in a few patients [7,10]. The physical examination of the current case revealed a firm, nodular, and symmetrically diffused neck mass.

Neck USG and CT are crucial in evaluating and assessing thyroid gland disorders [7,9,10]. Even though these diagnostic modalities can detect adipocytes in the thyroid gland, the particular diagnosis is achieved by histopathological study of the surgically resected specimen. In the presented case, thyroid gland adiposity was detected on the CT (the CT findings mentioned in the case presentation section).

The number of cases demonstrated the role of FNA and FNAC in the thyrolipoma diagnosis besides the pathological studies [7,10,11].

Surgical resection of the thyroid gland through total thyroidectomy was performed on our patient and the definitive diagnosis of thyrolipoma was achieved by the histopathological study of the surgically resected specimen.

## 4 Conclusion

Due to its rarity, fat-containing thyroid lesions offer a diagnostic dilemma in the surgical field. The low sensitivity and specificity of imaging studies are aided by FNA and FNAC help in diffuse thyroid lipomatosis diagnosis. However, the gold standard in identifying thyrolipoma is histologically after the thyroidectomy procedure. In such situations, thyroid tissue enlargement rate and dimensions are essential parameters used to assess tissue's malignancy tendency. Our case report emphasizes the diagnostic and surgical challenges of diffuse thyroid lipomatosis.

## Consent

Written informed consent was obtained from the patient to publish this case report and accompanying images. On request, a copy of the written consent is available for review by the Editor-in-Chief of this journal.

## Provenance and peer review

Not commissioned, externally peer-reviewed.

## Ethical approval

Ethical approval is not applicable. The case report is not containing any personal information. The ethical approval is obligatory for research that involve human or animal experiments, so there is no institution that waived ethical approval.

## Funding

No funding or grant support.

## Author contribution

Saqer Alenezi: literature review, paper writing, editing, and picture editing.

Athary Saleem: paper writing, editing, picture editing, manuscript drafting.

Omar Alhajri: paper editing.

Ous Alozairi: performed surgery, paper editing, and supervision.

## Guarantor

Saqer Alenezi, M.D., General Surgery Department, Al-Adan Hospital, Kuwait.

Email: saqeralenezi96@gmail.com

## Research registration number

Not applicable.

## Conflict of interest statement

There are no conflicts of interest to declare by all the authors.

## References

[1] Himmetoglu C., Yamak S., Tezel G.G. (2007). Diffuse fatty infiltration in amyloid goiter. Pathol. Int..

[2] Lo R., Donaldson C. (2013). Diffuse lipomatosis of the thyroid gland. Ultrasound Q..

[3] Esin Çelik Z. (2015). Mature fat containing thyroid lesions. Eur. J. Gen. Med..

[4] Gnepp D.R., Ogorzalek J.M., Heffess C.S. (1989). Fat-containing lesions of the thyroid gland. Am. J. Surg. Pathol..

[5] Lau E., Freitas P., Costa J., Batista R., Máximo V., Coelho R., Carvalho D. (2015). Loss of mitochondrial SDHB expression: what is its role in diffuse thyroid lipomatosis?. Horm. Metab. Res..

[6] Agha R.A., Franchi T., Sohrabi C., Mathew G., for the SCARE Group (2020). The SCARE 2020 guideline: updating consensus Surgical CAse REport (SCARE) guidelines. Int. J. Surg..

[7] Gamra O.B., Romdhane N., Nefzaoui S., Mahjoubi M., Abid W., Koubaa W., Mbarek C.C. (2016). Diffuse lipomatosis of the thyroid gland. Egypt. J. Ear Nose Throat Allied Sci..

[8] Hijazi D.M., Addas F.A., Alghanmi N.M., Marzouki H.Z., Merdad M.A. (2018). An enlarged goiter presenting with a rare diffuse lipomatosis of the thyroid gland. Am. J. Case. Rep..

[9] Gill M., Munjal G., Pawaria P., Momin Z.C., Gupta S., Saklani B., Sen R. (2020). Diffuse lipomatosis of thyroid masquerading as nodular goitre. Int. J. Health Sci. Res..

[10] Bell S., Sosa G.A., del Valle Jaen A., Picasso M.F.R. (2016). Thyroid lipomatosis in a 36-year-old patient with rheumatoid arthritis and a kidney transplant. Endocrinol. Diabetes Metab. Case Rep..

[11] Borges A., Catarino A. (2002). Case 53: adenolipoma of the thyroid gland. Radiology.

